# Case Report of a Large Pigmented Epithelioid Melanocytoma With Suspected Lymph Node Metastases

**Published:** 2016-12-16

**Authors:** Rie Akita, Yoshihiro Sowa, Daiki Morita, Toshiaki Numajiri

**Affiliations:** Departments of Plastic and Reconstructive Surgery, Graduate School of Medical Sciences, Kyoto Prefectural University of Medicine, Kyoto, Japan

**Keywords:** pigmented epithelioid melanocytoma, lymph node metastases, melanocytic tumor, melanoma, epithelioid blue nevus

## Abstract

**Objective:** Pigmented epithelioid melanocytoma was proposed by Zembowicz et al in 2004 including lesions previously diagnosed as human animal-type melanoma and epithelioid blue nevus. Tumors are composed of epithelioid and spindled melanocytes with heavy pigmentation. **Methods:** We report a case of pigmented epithelioid melanocytoma in 47-year-old Asian woman. The tumor sized 10 × 6 × 2 cm on the anterior aspect of right lower leg. On resecting surgery, we also found black pigmented lymph nodes in the groin area on harvesting skin graft. **Results:** Histological examination showed a typical figure for pigmented epithelioid melanocytoma. In groin area, melanocytes were observed only in the lymph node capsule. Thus, we conclude that melanocytes in the capsule of this case could not be associated with metastasis. The patient is doing well 2 years after the surgery, with no evidence of recurrence or distant metastases. **Conclusions:** Pigmented epithelioid melanocytoma is a rare, heavily pigmented, borderline, melanocytic tumor with infiltrative borders. It has potential for frequent metastases but shows an indolent clinical course.

Pigmented epithelioid melanocytoma (PEM) is a nosological group proposed by Zembowicz et al^1^ in 2004 that includes lesions previously diagnosed as human animal-type melanoma and epithelioid blue nevus. Traditionally, melanocytic neoplasms are categorized as either melanoma or melanocytic nevi, but recently some borderline melanocytic tumors have been described.^2-4^

PEM is a comparatively rare, densely pigmented, borderline, melanocytic tumor with infiltrative borders.^1^ The tumor has potential for metastases but shows an indolent clinical course.^1,5,6^ Here, we report a large mass diagnosed as PEM with suspected inguinal lymph node metastases.

## METHODS

**Figure 1:**
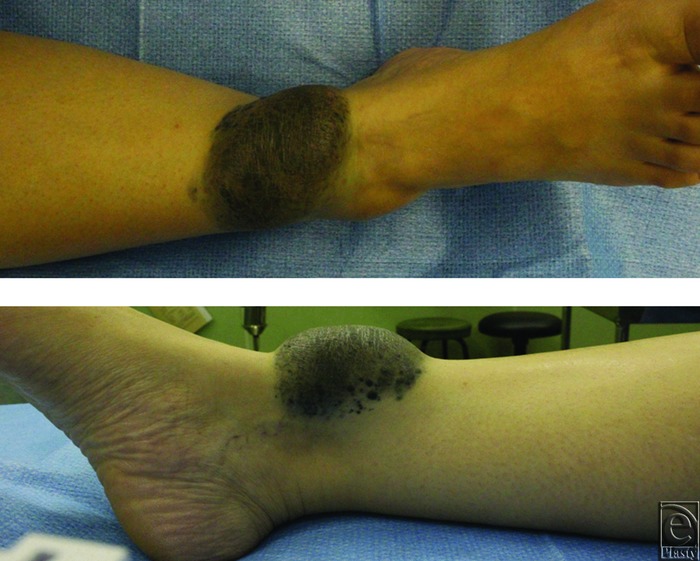
The overview of the tumor. A blue-gray dome-shaped soft mass sized 10 × 6 × 2 cm.

**Figure 2:**
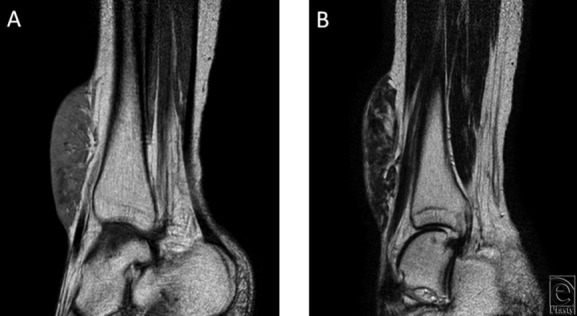
Magnetic resonance imaging findings. (a) T1 hypointensity and (b) T2 hyperintensity, irregular border on the deep side, and normal vessels and fat running through the mass.

A 47-year-old Asian woman presented with a long-standing history of a blue-gray dome-shaped soft mass sized 10 × 6 × 2 cm on the anterior aspect of her right lower leg (Fig 1). She and her mother stated that the mass has been observed since birth and had enlarged slowly with her growth. Inguinal lymph node swelling was not palpitated. Magnetic resonance imaging showed T1 hypointensity, T2 hyperintensity, and an irregular border on the deep side (Fig 2). Normal vessels and fat were present in the mass, but tumor invasion was not observed. The study was approved by the ethics committee of our hospital (Kyoto Prefectural University of Medicine).

**Figure 3:**
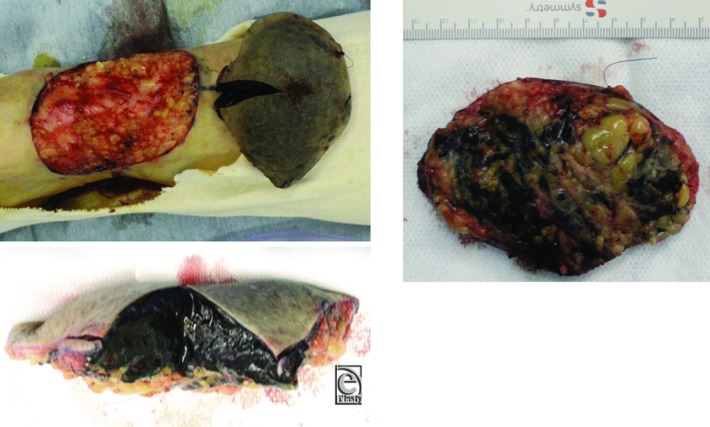
Resected specimen. The tumor was confined to subcutaneous tissue. The divided surface shows homogenous soft black tissue.

A marginal excision was performed. The cross-section surface of surgical specimen showed that the lesion was a homogeneous, soft, black tissue (Fig 3). On the deep aspect, small black-pigmented daughter lesions were scattered in the fat. We closed the wound using a full-thickness skin graft from the right groin area, where we found some black nodules thought to be lymph nodes in the subcutaneous tissue. We also resected these nodules for pathological examination, because we suspected them to be lymph node metastases.

## RESULTS

**Figure 4:**
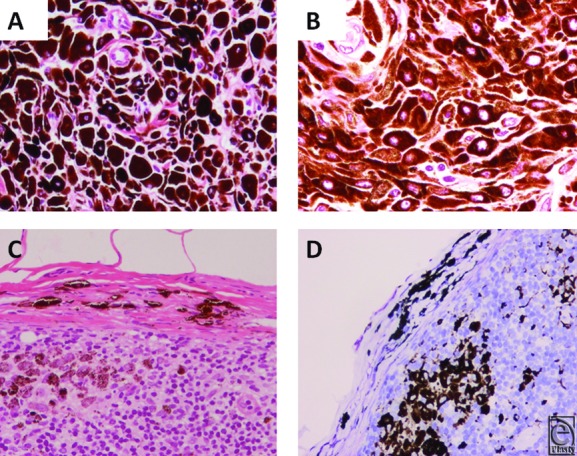
Microscopic findings. (a) Epithelioid cells are medium-sized round- to polygonal-shaped cells with densely pigmented cytoplasm, which contains round to oval vesicular nuclei with variably prominent nucleoli. H&E ×400. (b) In the peripheral area, epithelioid cells are intermixed with spindle cells. H&E ×400. (c) Lymph node specimen. Pigmented tumor cells are observed in the lymph node capsule. H&E ×100. (d) Lymph node specimen. Immunohistochemistry for CD68 shows that pigmented cells in the subcapsular space and in the lymph node parenchyma are not melanocytes but are melanophages. Giemsa ×100.

Histological examination revealed proliferation of densely pigmented epithelioid- and spindle-shaped cells in both the dermis and subcutaneous tissue. Breslow's tumor thickness was 22.4 mm, and Clark's level of invasion was level V. No tumor necrosis was observed (Fig 4).

Mitotic activity and severe nucleus/cytoplasmic atypia were not observed. In the lymph node, pigmented cells were observed both in the capsule and in the lymph node parenchyma (Fig 4*c*). Immunohistochemical stainings with S-100, Melan A, HMB45, and CD68 were performed. Although estimating immunohistochemical staining was difficult because of heavy melanin pigmentation, the leg lesion was seemed to melanocyte-derived cells that were positive for S-100, Melan A, and HMB45. The same melanocytes in the groin lymph node were observed only in the lymph node capsule and not in either the subcapsular space or in the lymph node parenchyma, whereas pigmented cells in the parenchyma were melanophages positive for CD68 (Fig 4*d*).

Considering the lack of malignant characteristics, we did not perform additional surgery. The patient is doing well 2 years after the surgery, with no evidence of recurrence or distant metastases.

## DISCUSSION

PEM is one of the blue nevus variants, including lesions previously diagnosed as the so-called animal-type melanomas and as epithelioid blue nevi. PEM occurs over a broad age range and has no ethnic predilection. These lesions also histologically overlap and can be found together with cellular blue nevi, malignant blue nevi, and other blue nevus variants.^1^

The pathological features of a PEM include densely pigmented, proliferating, dermal, melanocytic lesions with infiltrative borders. The tumors are composed of epithelioid- and spindled-shaped melanocytes. Mitotic activity is generally infrequent and ranges from 0 to 3 mitoses/mm^2^.^1^

We diagnosed the tumor as PEM because the histological findings are typical. Pigmented melanocytic cells in lymph node capsule itself is seen in cases of benign melanocytic nevi and not interpreted as evidence of metastasis.^1,7-9^ We concluded that the melanocytes in the lymph node capsule of this case were not associated with metastases.

To the best of our knowledge, the size of the lesion in our case (10 × 6 × 2 cm on physical examination and 22.4 mm in Breslow's tumor thickness) is large and rare for a PEM. The mean Breslow's thickness was 3.3 mm (1.4-10.5 mm) reported in the article by Zembowicz et al^1^ and 2.2 mm (0.80-10.00 mm) in the article by Mandal et al.^5^ In addition, a lesion sized 30 × 40 mm on the knee reported by Hayashi et al^10^ was the largest in the case reports that we found.

In the article by Zembowicz et al^1^, regional lymph node metastases occurred in 46% of the cases and liver metastasis in 1 case. The rate of regional lymph node metastasis in other studies of PEM has been much lower.^5,6^ Significance of sentinel lymph node biopsy in the patient with PEM remains unknown. Prognosis of PEM is apparently favorable, and clinical significance of sentinel lymph node metastases in PEM has not been studied.

We reported a large PEM in a 47-year-old woman with an indolent medical history. The finding of pigmented melanocytic cells was not an evidence of metastasis. This case could support the benign nature of PEM, yet detailed clinical and pathological analyses are still necessary for the management of PEM. We should carefully evaluate malignancy potential of tumors and avoid unnecessary invasive procedure.
